# Brain Training in Children and Adolescents: Is It Scientifically Valid?

**DOI:** 10.3389/fpsyg.2018.00565

**Published:** 2018-05-04

**Authors:** Teresa Rossignoli-Palomeque, Elena Perez-Hernandez, Javier González-Marqués

**Affiliations:** ^1^Department of Basic Psychology II, Complutense University of Madrid, Madrid, Spain; ^2^Department of Psychology and Education, Centro Universitario Cardenal Cisneros, Alcalá de Henares, Madrid, Spain; ^3^Department of Development and Educational Psychology, Autonomous University of Madrid, Madrid, Spain

**Keywords:** cognitive training, brain training, computer-based intervention, children, adolescents

## Abstract

**Background:** Brain training products are becoming increasingly popular for children and adolescents. Despite the marketing aimed at their use in the general population, these products may provide more benefits for specific neurologically impaired populations. A review of Brain Training (BT) products analyzing their efficacy while considering the methodological limitations of supporting research is required for practical applications.

**Method:** searches were made of the PubMed database (until March 2017) for studies including: (1) empirical data on the use of brain training for children or adolescents and any effects on near transfer (NT) and/or far transfer (FT) and/or neuroplasticity, (2) use of brain training for cognitive training purposes, (3) commercially available training applications, (4) computer-based programs for children developed since the 1990s, and (5) relevant printed and peer-reviewed material.

**Results:** Database searches yielded a total of 16,402 references, of which 70 met the inclusion criteria for the review. We classified programs in terms of neuroplasticity, near and far transfer, and long-term effects and their applied methodology. Regarding efficacy, only 10 studies (14.2%) have been found that support neuroplasticity, and the majority of brain training platforms claimed to be based on such concepts without providing any supporting scientific data. Thirty-six studies (51.4%) have shown far transfer (7 of them are non-independent) and only 11 (15.7%) maintained far transfer at follow-up. Considering the methodology, 40 studies (68.2%) were not randomized and controlled; for those randomized, only 9 studies (12.9%) were double-blind, and only 13 studies (18.6%) included active controls in their trials.

**Conclusion:** Overall, few independent studies have found far transfer and long-term effects. The majority of independent results found only near transfer. There is a lack of double-blind randomized trials which include an active control group as well as a passive control to properly control for contaminant variables. Based on our results, Brain Training Programs as commercially available products are not as effective as first expected or as they promise in their advertisements.

## Introduction

The use of new technologies is increasingly accepted in society, not only in educational settings and the general population, but also in the clinical field. More specifically, some “brain training” (BT) platforms, BT applications and BT video game-like products are becoming very popular. A rigorous evaluation of such applications is merited because most commercially available BT products have not been tested (Rabiner et al., 2010) despite being widely used. Traditionally, BT programs have been used mainly for relaxation therapy, as a tool to encourage self-control in children, or to restore abilities following brain damage. Furthermore, it specifically seems to have a special relevance for developmental psychopathology, being widely used, in disorders such as Attention deficit hyperactivity disorder (ADHD) (Rabipour and Raz, 2012), and in the elderly with cognitive decline (Papp et al., 2009). Despite this tradition, since Nintendo launched the BT game “How old is your brain?” in 2006, there has been increased interest in the general population in commercially available BT programs to improve, for instance, intelligence. Currently, BT is used both by the general population with typical development as well as in populations with neuropsychological impairment (Rabipour and Raz, 2012). In other words, “as we live in an increasingly technological society, the cognitive stimulation of healthy people requires more and more computerized resources” (Portellano, 2014, p. 136). Nevertheless, although BT is increasingly being marketed and aimed at the general population, it has a special use in neurologically impaired children and the elderly.

For this review, we must distinguish between different domains of BT or what researches refer to as computer-based interventions of “cognitive training” (CT). We must consider that the Eastern and European concepts differ; for instance, considering Tang and Posner (2009), we can classify attentional training (an example of CT) into two methods: the methods of the Asian tradition (for example mindfulness) and, the methods of the American or European tradition (such as BT programs). In the case of the first group, what is sought is to train a state of attention and self-regulation; while in the second, the aim is to alter specific brain processes related to cognitive tasks. In the case of American and European traditional methods, CT is based on the use of a repetition of exercises like those employed in cognitive psychology laboratories. This concept could be an extension of what we refer to as BT.

What is understood by “brain training?” BT is a program or activity which purports to improve a cognitive ability or general capacity by repeating certain cognitive tasks over a period of time. This is supposed to produce some changes in behavior, as well as at a neuroanatomical and functional level (Rabipour and Raz, 2012). Although this term is used mostly by companies rather than researchers (researchers commonly use “cognitive training”), BT refers to practicing core cognitive abilities with the goal of improving performance in other cognitive tasks (Simons et al., 2016). This model applies to computer-based programs as well as video game training or BT applications for touchscreens. Authors such as Nouchi et al. (2012) have researched the transfer effect of “video game training,” an issue commonly discussed in BT research, or McNab et al. (2015) who studied human cognition while using a touchscreen BT game-like application. For the present review, we have considered BT products supported by online or computer-based platforms, videogame-like products or applications for touchscreens.

To provide a better understanding of most BT research and BT efficacy, we refer to two concepts upon which most programs claim to be based: transfer and neuroplasticity. Neuroplasticity is supposed to alter neural connections and be reflected in the performance of cognitive skills or behavior, which is known as transfer or the transfer effect. Most researchers explain transfer effects due to neuroplasticity, but provide little data to support this idea. Here we are going to clarify both concepts.

### Transfer

Under this concept, authors such as Karbach and Unger (2014) distinguish between “near transfer” and “far transfer.” In the present review, we follow this distinction. The main goal of BT or CT should be not only to produce benefits in tasks similar to those directly trained or for the same construct, namely, near transfer (NT), but rather to benefit performance in other tasks that are different to those directly trained or for another construct: far transfer (FT). FT can have an impact on the user's daily life, and is understood as the “ecological” outcome of BT interventions.

Cognitive training interventions have shown FT even in relevant skills such as general intelligence (Raz and Buhle, 2006). In this line, Tang and Posner's (2009) study with adults seems to demonstrate that CT programs which target executive control or WM can benefit a wider variety of cognitive functions. In particular, in CT aimed at attention and WM, it seems that benefits could extend to fluid intelligence (Mackey et al., 2011). Klingberg (2010) explained this transfer as a result of the confluence of the prefrontal neural networks that support WM and fluid intelligence. Westerberg and Klingberg (2007) showed that practice in WM tasks gradually improved performance in WM tasks, and that the effect of practice also caused a general improvement in performance in a non-trained task such as a reasoning task. After training, WM-related brain activity was significantly increased in the middle and inferior frontal gyrus. According to this researcher, the changes could best be described by small increases in the extent of the activated cortex rather than activating additional areas. As we have seen, it is very common to justify transfer as a consequence of neuroplasticity. Strenziok et al. (2014) demonstrated FT of three cognitive training programs with healthy elderly people: (1) Brain Fitness (BF-auditory perception), (2) Space Fortress (SF-WM), (3) The Rise of Nations (RON-strategic reasoning). They found transfer of these trainings to other untrained areas (the first two), such as problem resolution of daily life and reasoning. The authors attributed their results to neuroplasticity, in that training produced changes in the integrity of gray matter in occipital-temporal areas (associated with improvement in problem solving of daily life), as well as in the ventral network. They hypothesized that this training produced changes in the attentional networks, leading to improvement in other processes. Some other studies in the adult population have tried to demonstrate the transfer effects of cognitive training though online platforms. Hardy et al. (2015) in a randomized controlled trial with a considerable sample (*N* = 4,715 fully evaluable participants) divided into two groups: CT group (general cognitive training through 49 games of the Luminosity online platform) and active control group (they completed crosswords puzzles) showed transfer effects. After training conducted at home (15 min per day over 5 days per week for 10 weeks), the cognitive training group showed greater improvements than controls in speed of processing, short-term memory, WM, problem solving, and fluid reasoning assessments, and greater improvements in self-reported measures of cognitive functioning, particularly in concentration compared to the control group, which could be considered as an ecological benefit of training. Nevertheless, the results of Hardy et al.'s study must be considered carefully because instruments of cognitive assessments, while based on other known tests, are part of the Luminosity framework.

Studies on typically-developing children also support the idea of near and far transfer of CT. Karbach and Kray (2009) aim to dilute the effectiveness of training cognitive flexibility through shifting tasks and its transfer to another untrained area. For this purpose, they conducted a trial using children (aged 7 to 9) and elderly people. The results showed that with only four training sessions of shifting (flexibility) tasks, positive results in the two types of transfer, NT and FT, were found in the trained group in inhibition, verbal, and visual WM and reasoning. In 9-year-old, typically developed children, Jaeggi et al. (2008) suggested that the transfer of the training program (WM training over fluid intelligence) depends on the gains obtained in the training: those that improved their performance notably in the trained task (an n-back, WM task - giving a response to a given sequence in a go/no go task) obtained better scores on intelligence tests [Test of Nonverbal Intelligence (TONI) and Raven's Standard Progressive and Matrices (SPM)], which suggests that good performance in CT leads to FT. In adolescents, Zinke et al. (2012), conducted a randomized controlled study with children aged 10–14 years, comparing the effectiveness of CT (task switching based on that used by Karbach and Kray, 2009), with the addition of physical exercise. In addition to evaluating transfer in similar tasks, they observed transfer to other untrained areas (inhibition, WM, and processing speed), concluding that both groups throughout the sessions significantly reduced the cost of change (time it takes them to shift set), as well as the number of errors (NT). They also improved WM and processing speed (FT).

In children with special educational needs, another study has found FT and long-term effects in children with brain damage. Galbiati et al. (2009) conducted a controlled trial of 6–18-year-old patients with severe brain damage which produced attentional deficits. The experimental group received BT stimulation in laboratory conditions consisting of 45-min sessions, 4 times per week for 6 months using three BT programs targeting attention (“Tabletop,” “Rehacom,” and “Attenzione e Concentrazione”). The results demonstrated significant differences in the trained group compared to controls in sustained attention and selective attention (they maintained attention longer and produced fewer omissions). In parental reports, those who were trained showed improvement in communication, daily life skills, and social skills; and those results were maintained at follow-up (12 months after intervention). In children with a low socioeconomic level (aged 7–8 years old), a combination of commercially available cognitive games and BT video game-like products (e.g., Rush hour, Professor Brainium's Games among others) have shown benefits in reasoning and processing speed (Mackey et al., 2011). In children with ADHD, many CT studies have been conducted, some of which seem to be effective in terms of NT and others in terms of FT. Kray et al. (2012), in a randomized trial, demonstrate that a relatively short cognitive training intervention (four training sessions in task shifting) on children aged 7–12 years with ADHD (medicated with methylphenidate), improved processes of inhibition and WM (components of executive function), but not fluid intelligence. Here we see lack of FT. In contrast, a randomized controlled trial (with children aged 6–18 years with ADHD) concluded that neurofeedback (NF), a type of CT, could be as effective as methylphenidate for treating the attentional and hyperactivity symptoms of ADHD, based on parental reports (Duric et al., 2012). According to Karbach and Unger (2014), the research on CT and ADHD seems to indicate that this training can compensate for deficits in executive functions (EF) and therefore improve school skills. Although this result has not been observed in all studies, this does not mean that the positive results are not encouraging. NF can be effective in relation to the improvement of EF, a key aspect of school performance (Illes and Sahakian, 2011).

### Neuroplasticity

Most BT programs claim to be based on brain neuroplasticity: the capacity that neurons have to modify their synaptic structures and form new neural connections (Pressler et al., 2011). There are studies that connect the practice of a certain activity to an increase in gray matter volume in the areas related to this activity. In a study in which adult participants learned to juggle, Driemeyer et al. (2008) concluded that changes in the gray matter can occur even after 1 week of training in a task; similar results were found by Scholz et al. (2009). Focusing on our area, to study neuroplasticity due to CT, researchers have focused especially on gray matter and neural activity changes. Some researchers, and especially BT developers, often relate changes in cognitive skills to neuroplasticity. Rabipour and Raz (2012) claim that due to brain plasticity, BT can alter attentional networks in the brain, and thus improve certain skills. In our view, to properly justify an association between cognitive skill improvements after training and neuroplasticity, neuroimaging techniques should be included in the trials.

In adults, studies focused on working memory (WM) training, such as Takeuchi et al. (2011) using a randomized controlled trial with young adults, demonstrated that a BT intervention, intensive adaptive training of WM using mental calculations (IATWMMC) was associated with a decrease in regional gray matter volume in the bilateral frontoparietal regions and the left superior temporal gyrus (neuroplasticity), and also with cognitive performance improving verbal letter span and complex arithmetic ability (transfer effect). Another study also found gray matter differences after undertaking WM training: in their pseudorandomized controlled trial, Caeyenberghs et al. (2016) studied a typical sample aged between 19 and 40 years, divided into two groups. The adaptive group trained WM at home using a Cogmed program (a computer-based program which aimed at WM and adjusted to user level, for 8 weeks with 45 min in each session, 40 sessions in total) vs. a non-adaptive group (training not adjusted to user level). Before and after training, cognitive assessment was applied, as well as white matter imaging techniques [diffusion tensor imaging (DTI)]. The results showed improvement in the adaptive group, not only in cognitive measures such as WM span, reasoning, and inhibition, but also changes in global integration based on white matter connectivity within a frontoparietal attention network. Another study with a similar design, related adaptive cognitive training to some changes in thickness of cortical structures (Metzler-Baddeley et al., 2016). In their pseudo-randomized study, an adult sample was divided into two groups: an active control group (who received training with no user-level adjustment) vs. adaptive training (for whom training was adapted to user-level performance); both groups trained using the Cogmed program. After training, neural changes were observed as increases in cortical thickness in some brain areas (right-lateralized executive regions) as well as reductions in others (such as the left pallidum). They related these changes in the brain to cognitive performance in near transfer assessment. These results support the idea of neuroplasticity due to a BT intervention. Apart from gray matter differences reflecting neuroplasticity due to CT, brain activity has been studied with the same purpose by means of the fMRI technique. Westerberg and Klingberg (2007) conducted a trial with three young healthy adults. Brain activity was measured on two separate days with fMRI: before practice and one day after practice of a WM task (Cogmed program). fMRI was also conducted during WM task performance. After training, WM-related brain activity was significantly increased in the middle and inferior frontal gyrus. Whereas this study provides data to support neuroplasticity, it lacks transfer evidence to other cognitive skills. With the same technique, fMRI, Clemens et al. (2013), through a randomized, controlled study of young adults and showed that some brain areas were commonly activated for alertness and focus attention training (participants trained attention through Cogniplus: four sessions of alertness or four sessions of focus attention training). Moreover, BT and assessment activated common neural areas described in the literature. These data support neuroplasticity, but there is no evidence of any transfer effect to other cognitions or behavior.

Having established a connection between neuroplasticity and BT in adults, we must question whether a similar result may also be found in children and adolescents, whose brain functioning differs due to developmental factors. In the following results section, we will mention certain studies that have proven neuroplasticity through brain activation changes in the following areas: dyslexia in which BT produces changes in language skills as well as changes in brain activation (observed by fMRI) in areas that are normally activated during performance of linguistic tasks, as well as in compensatory areas (Temple et al., 2003); cancer survivors, BT has also shown reduction in the activation of areas related to WM and attention apart from improvements in cognitive skills (Conklin et al., 2015) and increased brain activation in some areas of the prefrontal cortex (Kesler et al., 2011a); using the same technique with ADHD children-teenagers, Stevens et al. (2016) found that, apart from effects on behavior, responsiveness of WM frontoparietal circuits and executive process-specific WM brain regions were altered by training. In Turner syndrome patients, apart from cognitive improvements, it seems that after treatment (Luminosity), bilateral parietal lobe activation increased and frontal-striatal and medial temporal activation decreased in the math task (Kesler et al., 2011b). Using MEG with typically-developing children, Barnes et al. (2016) showed how WM training (Cogmed) impacts networks in the brain related to this function (inferior temporal and frontoparietal cortex). The magnitude of task-related patterns of brain activity was significantly associated with previous findings observed in resting-state activity (Astle et al., 2015). Studies using EEG techniques, such as Johnstone et al. (2017) with children with ADHD, showed how neurofeedback (NF), a type of CT, can produce brain activity changes, indicating normalization of atypical EEG features with reduced delta and increased alpha activity after training. In adolescents with multiple sclerosis, Hubacher et al. (2015) found that performance gains after cognitive training (attention and WM training through the BrainStim program) were accompanied by increased activity in the WM network and changes in inter-network connectivity (fMRI). Taking this into account, we must ask ourselves what types of BT engender neuroplasticity and whether neuroplasticity produces some observational effects in cognition and behavior.

### BT current limitations

Despite this background, other researchers highlight the lack of evidence of FT in many BT products (Cortese et al., 2015). Despite the increasing popularity of these training tools, Karbach and Unger (2014) claim that their results are neither robust nor consistent, and the transferability of training-induced performance improvements to untrained tasks seems limited. It must be considered that if learning is specific to the trained ability, as is often the case with BT programs, there is little generalization in relation to related tasks in new environments, limiting the practical impact of such learning. It may be the case that other activities, such as video games, music, and athletic training, show a more reasonable generalized effect (Green and Bavelier, 2008). What is essential for BT products is to establish clear cognitive targets that may have an impact on the user's daily live. Therefore, for many BT programs, FT is more difficult to prove than NT (Simons et al., 2016), not only in clinical populations, but also in a healthy or typically-developing populations. Supporting this concept, a large randomized controlled online study with 11,430 participants aged 18–69 years using a BT program (a BT tool designed by BBC Lab UK to improve reasoning, memory, planning, visuospatial skills, and attention) did not show any transfer effect in untrained tasks, even if they were parallel to the trained ones (Owen et al., 2010). These limitations are commonly found in research both with adults and with children. An example of these limitations may be seen in the study by Roberts et al. (2016). These authors studied the impact of WM training (Cogmed program) on WM skills and academic outcomes (reading, math, and spelling scores as primary outcomes) in children aged 6–7 years with low WM. WM training had an impact on the 4 short-term and WM outcomes, but had no impact on academic outcome (FT), which means that only NT was found and that some of these training effect did not maintain benefits over time. Another study with children with low WM scores (Ang et al., 2015) showed that training, whether updating training or Cogmed training, did not have FT on math, and NT it was not lasting in the long term. Another limitation of computer-based interventions is that in the short term they often produce improvements in the trained processes (NT), however, there are difficulties in interpreting data because of study design limitations (e.g., lack of a control group), which restricts the possible interpretations of the results, and they usually do not show improvement maintenance beyond 6 months (Rabipour and Raz, 2012). In a review of 10 randomized controlled trials with older people, the authors concluded that apart from a limited transfer effect, there is a lack of sufficiently follow-up periods to validate long-term effects and a lack of active control groups in the research designs (Papp et al., 2009). Another common limitation seems to be sustainable effects. For these reasons, an updated review of BT research in children and adolescents is required, as well as a proper classification of available programs considering their scientific background for practical reasons. The objective of this paper is to classify BT products available for children and teenagers according to research found using BT as an independent variable and analyzing its effects in terms of neuroplasticity, NT, FT, and long-term effects.

## Method

### Inclusion criteria

Studies from psychological sciences and neuroscience were reviewed and then included or rejected based on their relevance. First, a study was considered relevant for our research if it was based on empirical data from the use of a BT program (as an independent variable not combined with other BT products) with children or adolescents (4–17.9 years old) and its effects on NT and FT and/or neuroplasticity. Feasibility, compliance, acceptability, or factors to better benefit BT studies were not included. Second, the use of BT had to be for cognitive training purposes (motor skill training or emotional competence training were excluded). Third, the training described in the article must be commercially available. Fourth, this paper takes into consideration computer-based programs for children developed since the 1990s. Finally, the selection was limited to include only printed and peer-reviewed material, such as articles in journals, edited books, and research reports.

### Search terms and databases

Searches were conducted from June 2015 until March 2017 with the filters: English, Humans, in the following electronic database: PubMed. Heading searches for the following areas were combined:

Search 1: 7648 results

Cognitive training
(or) Brain training
(and) Children

Search 2: 6,105 results

Cognitive training
(and)Working memory training
(or) Attention training
(and) Children

Search 3: 2,589 results

Cognitive training
(and)Language
(or) reasoning
(and) children

Search 4: 60 results

Neuroplasticity
(and) cognitive training
(and) children.

Searches on CT products websites were also conducted to screen commercially available products as well as to screen any other published research (available in Pubmed but not found in our database searches). Following the inclusion criteria, 70 articles were included in the results.

**Figure 1:**
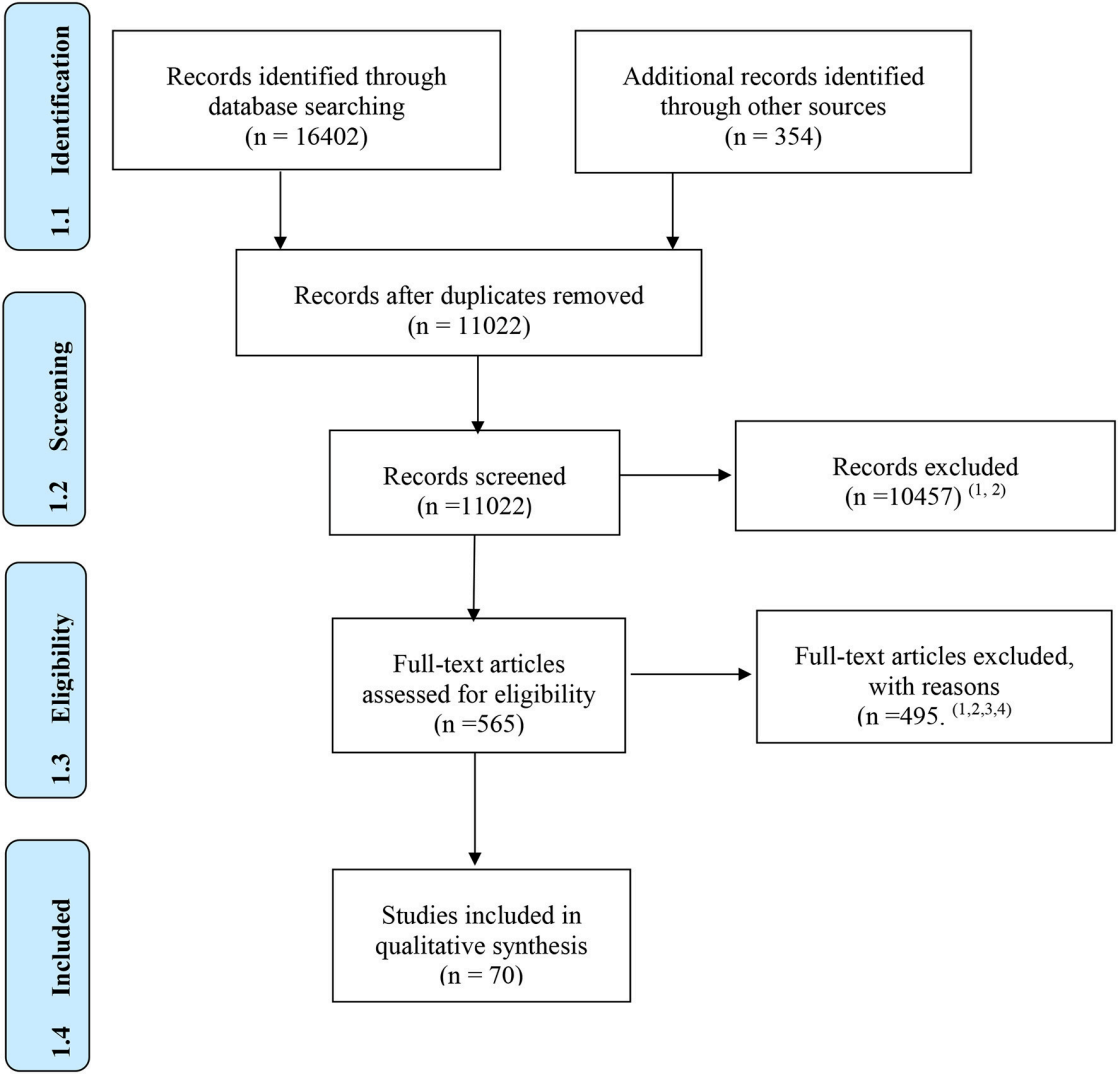
Reasons for exclusion: ^1^ independent variable is not a brain training product; ^2^ not targeted age; ^3^ products not commercially available; ^4^ feasibility studies. From Moher et al. (2009).

The selection flow diagram is shown in Figure 1.

### Method of analysis

Qualitative analysis was performed in this review. We established the following parameters to properly classify programs: Neuroplasticity, NT and FT, long-term effects, and study design.

In a first step, the different articles were read in order to determine whether they contained relevant information and whether they fulfilled the inclusion criteria. In a second step, for each selected article, the following information was extracted and entered into a table: study design, population and results (see Tables 1, 2). The information provided by the different studies was compared in order to explore program efficacy (see Table 3) and gaps or the future direction of BT research was included in the discussion section.

**Table 1:** Products supported by neuroscience research.

**Product name**	**Year release**	**Studies on children**	**Population**	**Design**	**Neuroimaging technique**	**Result**	**More information**
The Fast for Words	1993	Temple et al., 2003	Children with dyslexia. *N* = 20 aged 8–12 years old	Randomized Controlled trial (experimental vs. passive control) Non-independent	fMRI	Neuroplasticity Near and far Transfer	www.scilearn.com/results/research-independent-reviews
Teach-The-Brain	1999	Rueda et al., 2005	Typical developed children. *N* = 73 aged 4–6 years old	Randomized controlled trial (experimental vs. passive control) Follow up (2 weeks after final session)	EEG	Neuroplasticity Far transfer	www.teach-the-brain.org/learn/attention/index.htm
Cogmed	2001	Söderqvist et al., 2012	Typical developed children. *N* = 96 aged 4.0–4.5 years old	Pseudorandomized controlled Non-independent	DNA genotypes	Neuroplasticity Near transfer	www.cogmed.com/published-research
		Astle et al., 2015	Typical developed children. *N* = 33 aged 8–11 years old	Randomized controlled trial (adaptive vs. non-adaptive training group)	MEG	Neuroplasticity Near transfer	
		Conklin et al., 2015	Children survivors of cancer *N* = 68 aged 8–16 years old	Randomized single-blind controlled Follow up (6 moths)	fMRI	Neuroplasticity Near and far transfer	
		Barnes et al., 2016	Typical developed children. *N* = 33 aged 8–11 years old	Double-blind randomized controlled trial (adaptive vs. non-adaptive training group)	MEG	Neuroplasticity	
		Stevens et al., 2016	Children with ADHD *N* = 18 ADHD 18 non- ADHD controls aged 12–18 years old	Controlled trial	fMRI	Neuroplasticity Near and far transfer	
WinABC	2003	Penolazzi et al., 2010	Children with dyslexia *N* = 11	Interventional study	EEG	Neuroplasticity Near transfer	http://www.impararegiocando.it/WinABC50.htm
Luminosity	2007	Kesler et al., 2011a	Cancer survivors *N* = 23 aged 7–19 years old	A one-arm open trial pilot study	fMRI	Neuroplasticity Near transfer	www.lumosity.com/hcp/research/completed
		Kesler et al., 2011b	Turner Syndrome. *N* = 16 aged 7–14 years old	Case series study	fMRI	Neuroplasticity Near transfer	
Focus Pocus	2007	Johnstone et al., 2017	Children with ADHD *N* = 85 aged 7–12 years old	Randomized controlled trial Non-independent	EEG	Neuroplasticity Near and far transfer	www.focuspocushelp.weebly.com/focus-pocus.html

**Table 2:** Products derived from experimental and quasi-experimental trials.

**Product name**	**Year release**	**Studies on children**	**Population**	**Design**	**Result: type of transfer**	**More information**
Brain train (Captain's log)	1989	Rabiner et al., 2010	Children with attention difficulties. *N* = 77 first grade students	Randomized controlled trial Follow up (6 months after intervention)	Near and far transfer	www.braintrain.com/cognitive-training-research/
		Steiner et al., 2011	Children with ADHD. *N* = 41 middle school	Randomized controlled trial Follow up (6 months after intervention)	Far transfer	
		La Marca and O'Connor, 2016	Children with ADHD *N* = 5 aged 9–10 years old	Multiple-baseline-across-participants single-case model Follow up (5 months after intervention)	Near transfer	
Cogmed	2001	Klingberg et al., 2002	Children with ADHD *N* = 14 aged 7–15 years old	Double-blind controlled (adaptive vs. non-adaptive training group). Non-independent	Near and far transfer	www.cogmed.com/published-research
		Klingberg et al., 2005	Children with ADHD *N* = 53 aged 7–12 years old	Randomized controlled trial Non-independent	Far transfer	
		Thorell et al., 2009	Typical developed children *N* = 65 aged 4–5 years old	Randomized controlled Non-independent	Near and far transfer	
		Holmes et al., 2009	Children low WM *N* = 37 aged 9–10 years old	Controlled (adaptive vs. non-adaptive training group) Follow up (6 moths)	Near and far transfer	
		Holmes et al., 2010	Children with ADHD *N* = 25 aged 8–15 years old	Comparative study (not controlled not randomized) Follow up (6 moths)	Far transfer Long-term effects	
		Beck et al., 2010	Children with ADHD *N* = 52 aged 7–17 years old	Controlled Follow up (4 moths)	Far transfer Long-term effects	
		Mezzacappa and Buckner, 2010	Children low SES *N* = 9 Aged 8–10.5 years old	Pilot study single-group design with pre–post comparisons	Near and far transfer	
		Gibson et al., 2011	Adolescents with ADHD *N* = 47 aged 11–16 years old	Randomized controlled	Near transfer	
		Roughan and Hadwin, 2011	Children with behavioral difficulties *N* = 17 aged 11–13 years old	Randomized controlled Follow up (3 moths)	Near transfer Long-term effects	
		Kronenberger et al., 2011	Children with cochlear implant *N* = 9 aged 7–15 years old	Pilot study. 2 periods: wait and training Follow up (6 moths)	Far transfer Long-term effects	
		Løhaugen et al., 2011	Children preterm *N* = 46 aged 14–15 years old	Controlled trial	Near transfer Long-term effects	
		Bergman-Nutley et al., 2011	Typical developed children *N* = 101 aged 4 years old	Double-blind, randomized, controlled Non-independent	Near transfer	
		Dahlin, 2011	Children with special needs *N* = 57 aged 9–12 years old	Controlled trial Follow up (7 moths)	Far transfer Long-term effects	
		Green et al., 2012	Children with ADHD *N* = 26 aged 7–14 years old	Double-blind randomized controlled (adaptive vs. non-adaptive training)	Near and far transfer	
		Soderqvist et al., 2012	Children with low IQ *N* = 41 aged 6–12 years old	Pseudorandomized Follow up (1 year) Non-independent	Slightly far transfer on girls	
		Gibson et al., 2012	Typical developed children *N* = 31 aged 9–16 years old	Randomized controlled trial	Near transfer	
		Soderqvist et al., 2012	Children with low IQ *N* = 41 aged 6–12 Years old	Pseudorandomized and controlled (adaptive vs. non-adaptive training group) Follow up (1 year after training) Non-independent	Slightly far transfer on girls	
		Egeland et al., 2013	Children with ADHD *N* = 67 aged 10–12 years old	Randomized controlled trial Follow up (8 moth after intervention)	Near and far transfer Long-term effects	
		Hovik et al., 2013	Children with ADHD *N* = 67 aged 10–12 years old	Randomized controlled trial Follow up (8 moth after intervention)	Near transfer Long-term effects	
		Dahlin, 2013	Children with attention difficulties. *N* = 57 aged 9–12 years old	Controlled trial Follow up (approximately 7 months after intervention)	Near and far transfer Long-term effects	
		Dunning et al., 2013	Children with low WM *N* = 47 aged 7–9 years old	Double-bling randomized controlled trial Follow up (6 and 12 months after intervention)	Near transfer Long-term effects	
		Hardy et al., 2013	Children survivors of cancer *N* = 20 aged 8–16 years old	Pilot study randomized Follow up (3 moths)	Near and far transfer	
		Bennett et al., 2013	Children with down syndrome *N* = 21 aged 7–12 years old	Randomized controlled Follow up (4 moths)	Near transfer Long-term effects	
		Grunewaldt et al., 2013	Children preterm *N* = 20 aged 5–6 years old	Stepped Wedge randomized trial design	Near and far transfer	
		Holmes and Gathercole, 2014	Children low WM *N* = 72 aged 8–11	Randomized controlled trial	Near and far transfer	
		Foy and Mann, 2014	Children from economically disadvantaged communities. *N* = 50 aged 4–5 years old	Randomized controlled trial	Near and far transfer	
		Bergman-Nutley and Klingberg, 2014	Typical developed children *N* = 304 aged 7–15 years old Children with ADHD *N* = 176 aged 7–15 years old	Controlled trial Non-independent	Far transfer	
		Chacko et al., 2014	Children with ADHD *N* = 85 aged 7–11 years old	Randomized controlled trial (adaptive vs. non-adaptive training)	Near transfer	
		Dongen-Boomsma et al., 2014	Children with ADHD *N* = 51 aged 7–12 years old	Triple-blind, randomized, placebo-controlled study (adaptive vs. non-adaptive training)	Near transfer	
		van der Donk et al., 2015	Children with ADHD (Children with comorbid learning disabilities (LDs) and/or oppositional defiant disorder (ODD) were also included. *N* = 100 aged 8–10 years old	Randomized controlled trial Follow up (6 months after intervention)	Near transfer Long-term effects	
		Holmes et al., 2015	Children with specific language impairment *N* = 179 aged 8–11 years	Not controlled trial	Near transfer	
		Söderqvist and Nutley, 2015	Typical developed children. *N* = 42 aged 9–11 years old	Controlled trial Follow up (2 years after intervention) Non-independent	Far transfer Long-term effects	
		Ang et al., 2015	Children with low WM *N* = 111 aged 7 years old	Controlled trial Follow up (1 year after training)	Near transfer	
		Partanen et al., 2015	Children with special needs *n* = 64 aged 8–9 years old	Randomized and controlled trial	Near transfer (better results in combination treatment)	
		Kerr and Blackwell, 2015	Children with epilepsy n = 77 aged 5-−15 years old	Randomized controlled trial	Near transfer	
		Phillips et al., 2016	Children with brain damage *N* = 23 aged 8–15 years old	Double-blind randomized controlled (adaptive vs. non-adaptive training) Randomized controlled trial Follow up (3moths)	Far transfer Long-term effects	
		Fälth et al., 2016	Typical developed children. *N* = 32 first grade of primary school	Controlled trial Follow up (7 months after intervention)	Far transfer	
		Grunewaldt et al., 2016	Children preterm *N* = 37 aged 5–6 years old	Pilot study Not controlled Follow up (1 year after training)	Near transfer Long-term effects	
		Eve et al., 2016	Children with brain damage *N* = 7 aged 10–16 years old	Randomized Follow up (6 months after training)	Near transfer	
		Graziano and Hart, 2016	Children with behavioral problems *N* = 45 pre-schoolers	Randomized trial Follow up (6 moths)	Far transfer (better results in combination treatment)	
		Lee et al., 2016	Children preterm *N* = 12 preterm *N* = 10 term-born Aged 4–6 years old	Intervention study	Near transfer	
		Bigorra et al., 2016	Children with ADHD *N* = 66 aged 7–12 years old	Double-blind randomized controlled (adaptive vs. non-adaptive training) Follow up (6 moths)	Near and far transfer Long-term effects	
		Hadwin and Richards, 2016	Adolescent with T-score > 50 on anxiety test *N* = 40 aged 11–14 years old	Randomized controlled Follow up (4 moths)	Near and far transfer Long-term effect	
		Roberts et al., 2016	Children with low WM *N* = 452 aged 6–7 years old	Randomized controlled Follow up (1 year and 2 years)	Near transfer Long-term effects	
		Fuentes and Kerr, 2017	Children with epilepsy *N* = 28 aged 6–15 years old	Exploratory analysis Follow up (3 moths)	Near transfer Long-term effects	
		Hitchcock and Westwell, 2017	Typical developed children *N* = 148 aged 12 years old	Cluster-randomized, controlled trial (adaptive vs. non-adaptive training vs. passive control) Follow up (3 moths)	Near transfer (to similar task trained not to WM construct)	
		Conklin et al., 2017	Children survivors of cancer *N* = 68 aged 9–14 years old	Randomized, single-blind controlled Follow up (6 moths)	Near and far transfer Long-term effects	
Focus Pocus (Neurocog)	2007	Johnstone et al., 2012	Children with ADHD *N* = 128	Randomized controlled trial Follow up (6 months after intervention Non-independent	Far transfer Long-term effects	www.focuspocushelp.weebly.com/focus-pocus.html
Play Attention	2010 (recent version)	Steiner et al., 2011	Children with ADHD *N* = 41 Aged 7–11 years old	Randomized controlled trial Follow up (6 months after intervention)	Far transfer	www.playattention.com
		Steiner et al., 2014	Children with ADHD *N* = 104 aged 7–11 years old	Randomized controlled trial Follow up (6 months after intervention)	Far transfer Long-term effects	
Braingame Brian	2010	Dovis et al., 2015	Children with ADHD *N* = 89 aged 8–12 years old	Double-blind Randomized Placebo controlled trial Follow up (3 months after intervention)	Near transfer	http://www.gamingandtraining.nl/beschrijving-braingame-brian/
ACTIVATE™	2011	Bikic et al., 2015	Children with ADHD *N* = 122 aged 6–13 years old	Randomized controlled trial Follow up (3 and 6 months after intervention)	Near transfer	http://www.c8home.com/
SIGUEME	2013	Vélez-Coto et al., 2017	Children with autism disorder *N* = 74 aged 3–16 years old	Controlled trial	Near transfer	http://www.proyectosigueme.com/
Tali Program	2017	Kirk et al., 2017	Children with intellectual and developmental disability *N* = 76 aged 4–11 years old	Randomized double-blind placebo controlled trial Follow up 3 (3 months after intervention). Non- independent	Improvements at 3 months but not significant	https://www.monash.edu/medicine/research/what-is-the-tali-attention-training-program

**Table 3:** Scientific validity of Brain training programs for children based on Mahncke and Merzenich (2015).

	**Has the product demonstrated transfer of training to other laboratory tasks that measure the same cognitive construct as the training task?**	**Has the product demonstrated transfer of training to relevant real-world tasks?**	**Has the product performance been evaluated using an active control group whose members have the same expectations of cognitive benefits as do members of the experimental group?**	**How long are the trained skills retained?**	**Have the purported benefits of the training product been replicated by research groups other than those selling the product?**
Brain Train	Yes	Yes		6 months	Yes
The Fast for Words	Yes	Yes			
Teach-The-Brain	Yes	Yes		2 weeks follow up	yes
Cogmed	Yes	Yes	Yes, considering non- adaptive training as active control	2 years (follow up available 7–24 moths)	Partially, it counts with non-independent research
WinABC	Yes				Yes
Luminosity	Yes	Yes			Yes
Focus Pocus	Yes	Yes		6 months	
Play Attention		Yes		6 months	
BrainGame Brian	Yes		Yes		Yes
ACTIVATE	Yes				Yes
Sigueme	Yes		Yes		Yes
Tali program	No				No

## Results

After searching for results, we selected 70 articles which met with inclusion criteria. Then, we classified different commercially available BT programs for children according to their scientific background. Tables 1, 2 summarizes the main research of programs selected for this article.

We have classified BT programs as follows: (1) products supported by neuroscience research: computer-based programs in which neuroimaging techniques, such as fMRI, MEG, EEG etc., have been applied to prove program impact in terms of neuroplasticity; (2) Products derived from experimental and quasi-experimental trials: computer-based programs in which psychometric tests have been applied to test program impact. Finally, to further clarify the scientific validity of the programs, we have taken into account Mahncke and Merzenich's (2015) considerations about how to evaluate a BT program. This consideration includes questions related to program efficacy, study design, and long-term effects. Based on these criteria, Table 3 summarizes the scientific validity of the programs mentioned in the present paper.

### Products supported by neuroscience research

In this section, we included computer-based programs aimed at research which use neuroimaging techniques such as fMRI, MEG, EEG, DNA analysis etc., to prove program impact under neuroplasticity parameters.

Table 1 shows a summary of characteristics of each research based on the aforementioned programs.

#### Fast ForWord® (FFW)

This program is supported by independent research based on neuroimaging techniques for dyslexia (Temple et al., 2003). These authors have shown that people with dyslexia show dysfunction in phonological processing. FFW was applied to children with dyslexia (divided into an experimental and control group); after an average of 27.9 training days (100 min in 5 sessions per week), participants showed improvements in reading and oral language, pseudo-word decoding and comprehension, as well as changes in brain activation (observed by fMRI) in areas that are normally activated during performance of phonological tasks as well as in compensatory areas (left temporoparietal regions, left frontal inferior rotation, right hemisphere temporal and frontal regions, and the anterior cingulate gyrus). This suggests that this program alleviates dysfunctions associated with phonological processing, as well as producing compensatory activation in other areas.

#### Teach-the-brain

This program is based on independent research in neuroimaging techniques that measure brain activity though EEG (Rueda et al., 2005). It shows that 4–6-year-olds can improve EF and even intelligence quotient (IQ) after only 5 days of BT (with the aim of training the three attentional networks proposed by Posner and Petersen, 1990). They evaluate this evolution with EEG and psychologically-validated tests (Child ANT, Kaufman's brief intelligence test) and parent questionnaires, and conclude that, despite the genetic load on attention and executive functions, training produces improvements in these skills.

#### Cogmed

This program implements research based on neuroimaging techniques which measure brain activity in adults through fMRI (Westerberg and Klingberg, 2007), EEG (Liu et al., 2016), and DTI (Caeyenberghs et al., 2016). Specifically, for children, there are research studies that use neuroimaging techniques such as MEG (Astle et al., 2015; Barnes et al., 2016), fMRI (Conklin et al., 2015; Stevens et al., 2016), and DNA genotype (Söderqvist et al., 2012).

Astle et al. (2015) wanted to figure out whether WM training had an impact on brain connectivity at rest in those areas typically associated with WM and controlled attention as well as in cognitive tests. Typically developed children, aged 8–11 years, completed 20 sessions of computerized WM training at home. Before and after the training, all of the children underwent a 9-min resting state (MEG) scan and completed standardized assessments of short-term and WM. The results showed that the adaptive group (in which the training was adapted to user execution) demonstrated significant improvements in standardized scores in the untrained short-term and WM assessments. Adaptive training enhanced resting functional connectivity: significant enhancement of connectivity was found in the bilateral frontoparietal network, superior parietal cortex, and a portion of inferior temporal cortex. Moreover, connectivity changes associated with training were greatest in those who displayed the greatest improvement in WM capacity.

Using MEG, Barnes et al. (2016), showed how this CT program impacted networks in the brain related to WM, specifically on frontoparietal and temporal brain structures. In this study on typically developed children, WM training involved at least 20 training sessions (35 min) for 4–6 weeks at home. WM task-related MEG data were collected before and after the training intervention. After the intervention, researchers identified “significantly increased cross-frequency phase amplitude coupling in children who completed training, specifically between the upper alpha rhythm (at 16 Hz), recorded in superior frontal and parietal cortex with high gamma activity (at ~90 Hz) in inferior temporal cortex” (Barnes et al., 2016 p. 1). Thus, it seems that BT can modulate brain waves. The authors associated this altered neural network activity with cognitive skill enhancement. Furthermore, the magnitude of task-related coupling found in this study (as a pattern of brain activity) is significantly associated with previous findings observed in resting-state activity (Astle et al., 2015). In addition, the results showed that changes in frontoparietal to inferior temporal phase amplitude coupling were significantly predictive of children's improved performance in the WM task; in this case, there is evidence of a relationship between neuroplasticity and cognitive performance.

Through the fMRI technique, Stevens et al. (2016) conducted controlled trials comparing18 children with ADHD to 18 control subjects aged 12–18 years. After training (standard Cogmed protocol: 5 weeks and 25 sessions with 30–40 min per session), the trained group showed some NT and FT (less ADHD clinical symptoms reported by parents). The responsiveness of both WM frontoparietal circuits and executive process-specific WM brain regions was altered by WM training. Within the same neuroimaging technique, Conklin et al. (2015), in a randomized controlled trial on children survivors of cancer, proved that Cogmed training affects cognition and brain activity (5–9 weeks with 25 sessions of 30–40 min at home). After training, NT was found in WM and FT (attention and processing speed) as well as brain activity changes: reduction in activation of left lateral prefrontal and bilateral medial frontal areas related to WM and attention.

Finally, we found a DNA genotype study (Söderqvist et al., 2012) which examined the effects of polymorphisms in five genes involved in dopaminergic pathways after CT: WM training, Non-verbal reasoning training (NVR) or a combination, in preschoolers though a pseudorandomized controlled trial. They conducted 25 sessions of 15 min per day at home. WM training produced NT, and NVR produced gains in fluid intelligence. With regard to neuroplasticity, the authors found that polymorphisms of the DAT1 gene were associated with training effects: variation in the dopamine transporter gene (DAT1) influenced improvements in WM and fluid intelligence.

#### WinABC program

WinABC is a computer-based program developed to improved literacy skills, supported by a study which supports NT and neuroplasticity in children with dyslexia (Penolazzi et al., 2010). In their study, 11 children with dyslexia aged 9–11 years received 6 months of phonological training at home (5 times a week for 10 min per day). Besides NT, the authors found that those children who had the greatest reading speed enhancement showed the largest left posterior EEG beta power increase in phonological task execution after the training sessions. Nevertheless, as this study is an intervention study (not controlled), the result must be considered with caution.

#### Luminosity

This program is based on research on neuroimaging techniques using fMRI in children with cancer or Turner syndrome (Kesler et al., 2011a,b), as well as EEG studies in adults (Schneider et al., 2013).

This program was found to be effective in training EF with children who have suffered cancer. Kesler et al. (2011a) designed a home cognitive training program (8 weeks of intervention/5 session per week/20 min per session). Not only cognitive assessment at baseline and post intervention were applied, but also fMRI measures were made. Following the cognitive intervention, participants showed a significant increase in processing speed, cognitive flexibility, verbal, and visual declarative memory scores, as well as a significant increase in pre-frontal cortex activation compared to the baseline (inferior, middle, and superior frontal gyrus activation). Nevertheless, in this study there was no correlation between cognitive scores at post-intervention and brain activation in fMRI.

Luminosity seems to be effective for children with Turner syndrome who have low math abilities. Kesler et al. (2011b) assess some mathematical skills and other involved mental processes (processing speed, attention, cognitive flexibility) as well as brain activation before and 1 week after training. The training consists of an adaptive BT program focused on number sense and general problem-solving skills (5 sessions/6 weeks/20 min per session, at home). After training, the participants significantly improved their basic math skills, including number sense and calculation, as well as processing speed, cognitive flexibility, and visual-spatial processing skills. In terms of brain activation, the participants showed significantly increased bilateral parietal lobe activation and decreased frontal-striatal and mesial temporal activation in math tasks. Nevertheless, it must be considered that a controlled randomized study in this field would contribute to contrasting or supporting this study which lacks a randomized controlled design.

#### Focus pocus

Focus Pocus is one of the BT programs based on neurofeedback (NF). NF is a process of learning in which the user is rewarded for positive brain activation modulation (Fox et al., 2005). The training consists in modulating brain waves to achieve a series of goals within a computer game. This program is based on empirical research using EEG records to demonstrate neuroplasticity due to training. Johnstone et al. (2017), in a controlled randomized study, showed how neurofeedback training (at home) can produce brain activity changes, indicating normalization of atypical EEG features with reduced delta and increased alpha activity after training in children with ADHD.

### Products derived from experimental and quasi-experimental trials

In this section, we include computer-based programs based on research using psychometric testing to evaluate program impact. Some of them have been included in the first section such as Cogmed.

Table 2 shows a summary of characteristics of each research project based on the different programs mentioned above.

#### Braintrain

Some randomized controlled studies have also been conducted using BrainTrain products (such as Capitain's Log) with ADHD children (Rabiner et al., 2010; Steiner et al., 2011, 2014).

A combination of CT with other techniques could also be of interest for children with ADHD symptoms (Rabiner et al., 2010). Cognitive training (“Capitain's Log”) and computer intervention that facilitates the understanding of instructions, or “Computer-assisted instruction,” entails a decrease in ADHD symptoms in the classroom, especially for those who initially showed more symptoms of inattention, after 28 sessions of 75 min with first grade children. Steiner et al. (2011) showed the effectiveness of two neuroscientific interventions in children with ADHD; a neurofeedback program (“Play attention”) and a computerized CT program (“Brain Train/Captain's Log”). After an average of 23.4 sessions in their schools, the parents reported a significantly greater improvement in symptoms associated with this disorder than in the control group. In subsequent studies, the same authors demonstrated that the effects were maintained at a 6-month follow-up (Steiner et al., 2014).

Finally, La Marca and O'Connor (2016) tried to determine whether neurofeedback training (“SmartMind Pro”) is effective at improving not only attention and executive functions, but also reading comprehension and fluency in children with ADHD Inattentive Subtype. The participants followed 40 NF sessions in a school environment and three measurements of each were obtained: baseline, post-test, and 5-month follow-up. The results showed that following the intervention, improvements were observed in a continuous performance test and a shifting attention task. The results obtained from reading fluency tests revealed little change, although participants demonstrated gains in reading comprehension. In this case, it would be interesting to conduct a randomized controlled trial that included attentional measures, in order to support their findings.

#### Cogmed

A study of typically-developing 4–5-year-old children was conducted by Thorell et al. (2009). The sample was divided into three groups: a group that received training in visuospatial WM (from Cogmed), another group that received inhibition training (through a go/no-go task), and a third, passive control group. After 5 weeks of training (they attended 15-min sessions each day), the children who received WM training improved significantly in non-trained visuospatial WM tasks, as well as in attention tasks (the children who were trained in inhibition did not display significant improvements in untrained tasks). In this case, Cogmed seemed to be effective for typically-developing children aged 4–5 years in terms of NT. In another study with typically developed children of the same age, Bergman-Nutley et al. (2011) demonstrated that Cogmed was effective for training WM in this population. First graders may also receive some benefits from CT (Fälth et al., 2016). In their study, children who received WM training (Cogmed standard protocol) showed significant improvements in a word decoding test compared to the control group. The implication is that there is a WM requirement for initial readers when the decoding process is not yet automatized, and the training was effective in improving this component. In another study with typically-developing children aged 9–11 years (Söderqvist and Nutley, 2015), it seems that WM training can have some FT on math and reading. An experimental group received 25 sessions for 20 min over 5 weeks at school, while a control group continued as usual. 12 months after training, the experimental group showed greater development in reading and math compared with a matched control group (maintained at a 2-year follow-up assessment). Furthermore, the progress in both math and reading in the trained group was directly related to the amount of improvement seen in the WM tasks. These results demonstrate transfer effects of training with a long-term effect. Nevertheless, these results must be considered with caution due to the non-independent nature of the study (the researchers have any kind of connection to the company or product). In children aged 9–16, Gibson et al. (2012) found that only the active maintenance of a limited amount of information in primary memory was improved by the program, however, no other WM components were improved. Finally, Hitchcock and Westwell (2017) compared WM training in children aged 12 years (adaptive vs. non-adaptive training) and passive control group, and did not find any transfer in task-related attention, reading, mathematics, or regulation of emotional, social, and behavioral challenges. It seems that studies on typically-developing children support evidence of NT (especially in preschoolers), yet there is no independent research to support FT for this population.

An early study of WM training effects on children with ADHD (Klingberg et al., 2002) showed that WM training produces improvements in trained capacities as well as reasoning, interference control and inhibition of motor skills after 5 weeks of training. Klingberg et al. (2005) showed that after training with the standard Cogmed protocol, the trained group obtained better results compared to the active control group in verbal WM, inhibition and abstract reasoning. Transfer in both studies is not only NT but also FT. However, these initial studies are not independent and therefore must be considered with caution. Another attempt to prove the benefits of behavioral ADHD symptoms (FT) through WM training has been conducted by Beck et al. (2010). In this controlled trial, the experimental group improved in the areas of inattention, the overall number of ADHD symptoms, initiation, planning, and WM as rated by parents. Teacher ratings approached significance at posttreatment and at a 4-month follow-up in the area of initiative. Green et al. (2012), in a double-blind randomized controlled trial, showed that WM training through standard Cogmed protocol, reduced off-task ADHD associated behavior (distractions during performance of tasks). Other studies, such as Dahlin (2013), relate WM training to school performance in math for an experimental group that received the Cogmed standard protocol. Compared to controls, the experimental group improved significantly in WM tasks and in math results. However, because the sample was not randomized, the results should be taken with caution. Egeland et al. (2013) demonstrated the effectiveness of the Cogmed program in improving processing speed in children with ADHD as well as improvements in math and reading. The experimental group's scores (after undergoing Cogmed standard training) significantly increased compared to the control group in visual and auditory WM. A later study conducted by Bigorra et al. (2016) showed that an adaptive training group, compared to the non-adaptive training group, significantly improved in WM, EF (as rated by parents and teachers), reduced impulsivity and ADHD symptoms; and those gains were maintained at a 6-month follow-up. Holmes et al. (2010) compared medication treatment for ADHD with Cogmed training. The results demonstrated that WM training produced WM and central executive gains that were maintained 6 months after treatment; nevertheless, this is a comparative study (not controlled). Despite these results using the same program on children with ADHD, van der Donk et al. (2015), did not find FT. In their study, one group received 5 weeks of cognitive training and another received a “care in class” treatment developed for the research. They valued not only cognitive outcomes and academic performance but also behavioral aspects (including after 6 months of intervention). The authors concluded that CT produced improvements at a cognitive level (in the different tests), but not in academic performance or behavior. In the same way, Chacko et al. (2014) found that WM training (Cogmed) produced benefits in WM, but not in behavior and academic achievement (FT). Similar results were obtained by Dongen-Boomsma et al. (2014) who found only NT, and in this case, it did not survive correction for multiple testing. Gibson et al. (2011) found NT after WM training in adolescents with ADHD. They conceptualize WM in two aspects: (1) retention and maintenance of information during distractions, and, (2) recovering information from the secondary memory (SM). Likewise, in a later study (Gibson et al., 2012), after modifying the exercises included in the standard version of Cogmed-RM from simple span to complex span, they did not find benefits on SM which is typically impaired in children and adolescents with ADHD. Their results showed WM training to be effective only for the first aspect of WM. In conclusion, there is some evidence to support Cogmed intervention in ADHD to obtain NT. FT results are controversial due to a lack of consistent findings, failures to replicate, and methodological limitations.

In children with low WM capability, Bergman-Nutley and Klingberg (2014) attempted to determine whether WM training (Cogmed standard protocol) could show FT on following instructions and arithmetic. They assessed WM (five times during and after training), following instructions and arithmetic using tests developed by Pearson and Cogmed. The training group improved significantly more than the control group in all three transfer tests. Using a regression model, transfer increased linearly with the amount of training time, and correlated with the amount of improvement on the trained tasks. It must be considered that this study is non-independent. Another study with low WM children aged 9–10 years was conducted by Holmes et al. (2009). The controlled trial results showed that adaptive WM training benefitted WM and mathematical reasoning, and those gains were maintained after 6 months. Holmes and Gathercole (2014), in a randomized controlled trial with children aged 8–11 with low academic achievement, showed that after WM training (Cogmed standard protocol conducted by teachers at school), WM, math and literacy improved. No follow-up was available. Along the same lines, Dunning et al. (2013) tried to demonstrate, through their randomized controlled study, the impact of CT (6 weeks of training) on WM, general intelligence, literacy and mathematics. The sample was divided in three groups (adaptive training, non-adaptive, and passive control group). The group who received adaptive training improved significantly in WM tests, maintaining this progress in visuospatial and verbal WM after 1 year. However, they did not obtain significant results in relation to the other groups in other cognitive areas (FT). In the same way, Ang et al. (2015) showed that training, whether updating training (seven computerized games were developed for the updating training: four games were based on the running span paradigm and three games were based on the keep track paradigm) or Cogmed training, did not show FT for math, and only NT which it was not maintained in the long term, beyond six months after training. Finally, the results of a study by Roberts et al. (2016) with low WM children demonstrated benefits in NT (only visuospatial short-term memory) which were maintained at 12 months. FT was not found in reading, spelling or math. In this population, robust findings supported NT and long-term effects (but not further than 6 months), while FT and longer-term effects were not replicated.

For children with low to moderate IQ, some partial benefits of training have been shown. A study with children with intellectual disability (IQ < 70), was conducted by Soderqvist et al. (2012): the sample was pseudorandomized in two groups (adaptive training vs. non-adaptive training) of WM (Cogmed standard protocol), and non-verbal training (NVR). 20 sessions were conducted at home (80% sample) or at school (20% sample). After training, the female participants showed improvement in instruction comprehension but not in other areas (reasoning, language, behavior rated by parents etc.) After a 1-year follow-up there were no significant improvements. It seems that individual differences compromised results: only female participants without an additional diagnosis and with higher baseline performance showed greater progress. In this sense, a minimum cognitive capacity seems necessary for the training to be beneficial, and a greater training time is required to reach sustainable training effects. Similar results were found in a pseudorandomized trial with children with low IQ (Soderqvist et al., 2012). A randomized controlled study on children with Down syndrome conducted by Bennett (Bennett et al., 2013) showed that WM training (Cogmed 10–16-week period at school; three times a week for 25 min per session), produces NT and the effects were maintained at a 4-month follow-up. Partanen et al. (2015) demonstrated in a randomized controlled trial that WM training in combination with metacognitive techniques produced a significant difference in WM maintained at a 6-month follow-up. No transfer to arithmetic or reading and writing skills occurred in any of the two training conditions. In this population, only Dahlin (2011) has found FT; a controlled trial showed that children trained in WM Cogmed standard protocol at school increased scores in reading comprehension, and those gains were maintained at a 7-month follow-up. Some variables, such as cognitive level in lower IQ children, might influence WM training effects, but few transfer benefits in WM and reading comprehension were found.

Focusing on children with language learning disabilities, Holmes et al. (2015) compared children diagnosed with Specific Language Impairment (SLI) to children with typical language performance. There was no control group and both groups received intervention. They took part in 20 sessions of 45-min over 8 weeks in small groups at school. The results showed that both groups improved their visuospatial short-term memory. However, the SLI group improved significantly more in one of two verbal STM measures (digit span). Exploratory analyses across the sample established that low verbal IQ scores were strongly and highly-specifically associated with greater gains in verbal span-like WM tasks, and those children with higher verbal IQs made greater gains in visuospatial STM following training. In another study, children with cochlear implants received the standard Cogmed protocol (Kronenberger et al., 2011). The researchers compared scores during wait time and training. After training, children demonstrated a significant improvement in measures of verbal and nonverbal WM, sentence-repetition skills and parent-reported working memory behavior. Sentence repetition continued to show marked improvement at a 6-month follow-up. In this area, randomized controlled trials would be crucial to replicate results.

A number of studies using Cogmed have been conducted with a population at risk of learning disabilities. On the one hand, some studies have focused on low birthweight or preterm children. Grunewaldt et al. (2013) conducted a stepped-wedge randomized trial with children aged 5–6 years who were born preterm. They showed that WM training (Cogmed JM version: 10–15 min per session for 5 days per week over 5 weeks at home) benefitted WM and auditory attention, phonological awareness, facial memory, narrative memory, spatial span, and sentence repetitions. There were no effects on anxiety reduction. Later, Grunewaldt et al. (2016) also studied the effects of WM training on children with the same characteristics. An experimental group received the standard Cogmed JM protocol at home. After training, some gains or equivalent scores as the control group were found in facial memory, narrative memory and spatial span, which remained at a 7-month follow-up. No group differences in performance gain were found for attention and behavior. It seems than FT to attention and behavior was not found in this case. A study conducted by Lee et al. (2016) on children aged 4–6 years did not find NT in preterm and normal-term children in WM after training (Cogmed JM version), and also found no FT to other domains such as attention and executive functions. Finally, a controlled trial on adolescents conducted by Løhaugen et al. (2011) showed that after training (standard Cogmed protocol) gains in WM were produced and maintained after 6 months, yet, no FT was evidenced. In this population, NT and FT in memory has been demonstrated, nevertheless there have been no findings so far for attention or behavior. On the other hand, children with a low sociocultural level (SES) are also at risk of potential learning difficulties. Foy and Mann (2014) carried out a study in an attempt to prevent learning difficulties. Through a sample of children aged 4–5 years (pre-readers) with a low socio-cultural level, they assessed whether WM training had some NT in WM, as well as FT on self-regulation and pre-literacy skills. For this purpose, one group received training in WM and another group did not receive any intervention. Their conclusions are that training favors the visuospatial memory of the trained children, as well as their self-regulation or executive control (assessed in inhibition tasks), but not on the prerequisites of literacy (e.g., phonological awareness or knowledge of letters). Another study on children with a low socioeconomic level was conducted by Mezzacappa and Buckner (2010). In this pilot study with a single group design, they compared WM and behavior (symptoms of ADHD before and after training as rated by teachers). After treatment, WM and behavior improved. Further research in this area is needed to provide more robust results.

Some researchers have focused on populations with different diseases such as cancer. Hardy et al. (2013) conducted a pilot study with child and teenage survivors of cancer. Immediately after treatment, the adaptive training group displayed significant improvements (not at follow-up) in their visual WM and in parent-rated learning problems, compared with those in the active control group. Conklin et al. (2017), in a randomized controlled trial with children aged 9–14 years, showed that after intervention, the trained group improved in WM, attention and processing speed. WM and processing speed gains were maintained at a 6-month follow-up. In this area, further research is required to better clarify the efficacy of Cogmed intervention. For children and adolescents with epilepsy, Kerr and Blackwell (2015) conducted a randomized controlled trial, the results of which showed that the trained group had significant post-interventive treatment effects for visual attention span, auditory WM, and visual-verbal WM (NT). Similar results were obtained by Fuentes and Kerr (2017), nevertheless FT (in fluid reasoning) was not observed. Indeed, further research is needed in this area to replicate results and to demonstrate the existence of any FT. Finally, in terms of brain damage, Eve et al. (2016) conducted a pilot study and a long-term follow-up with children who had suffered from an arterial ischemic stroke. They receive the standard Cogmed WM Training at home, supervised by their parents. Measures of WM, attention, and mathematical achievement were conducted before and after intervention, and at a 1-year follow-up. The results indicated that a significant improvement in phonological-loop WM was produced, however, this improvement was not maintained after 12 months. No additional significant improvements on standardized psychometric outcome measures were seen either immediately or at the 12-month follow-up. Phillips et al. (2016) compared adaptive vs. non-adaptive training in children with brain damage. The results demonstrated a significant difference in favor of the adaptive training group in WM and reading (reading comprehension and reading accuracy); the latter was maintained at a 3-month follow-up. However, no benefits were found in math. This finding may not support WM training for these patients; thus, further randomized controlled trials with children with brain damage would help to clarify this issue.

Finally, some studies have been conducted on children and adolescents with behavioral problems. Regarding children with externalizing behavior problems, Graziano and Hart (2016) conducted a randomized trial on preschoolers. In this study, the participants completed an 8-week intervention. They were allocated to one of three programs (STP-PreK = summer treatment program for pre-kindergarteners which involved BT (Cogmed), PT = parent training (parents were trained in some parenting techniques), and STP-PreK Enhance (which involved additional social skills, self-regulation strategies). The results suggested that, although all groups improved in behavioral functioning groups at a similar magnitude, children in the STP-PreK Enhanced group experienced greater growth over time. This group and STT-PREK maintained improvements at a 6-month follow-up in academic achievement, emotional knowledge, emotion regulation, and executive functioning compared to children with PT only. In children with behavior problems aged 11–13 years, Roughan and Hadwin (2011), in a randomized controlled trial, showed that the group trained in Cogmed (standard protocol) had better post-training scores in measures of IQ, inhibition, test anxiety, teacher-reported behavior, attention and emotional symptoms, compared with a non-intervention passive group; differences in WM were also evident at a 3-month follow-up. In adolescents with high scores on anxiety questionnaires, Hadwin and Richards (2016), in a randomized controlled trial, compared WM training (Cogmed standard protocol) vs. CBT intervention (small group activities on feelings, thoughts, relaxation techniques, problem solving, and coping strategies in small groups). After treatment, the WM training group showed significant gains in WM. Both groups reported fewer anxiety symptoms, demonstrated increased inhibitory control and a reduction in attentional biases to threat post intervention, and these results were maintained after 4 months. In children with behavioral problems, the results are encouraging for better regulation of behavior though cognitive training of WM.

#### Focus pocus

This program, mentioned in section 1, is also supported by a study using psychometric tests to improve training efficacy. Johnstone et al. (2012) showed, in children with ADHD, that the combination of CT (Focus Pocus exercises) with and without neurofeedback, and compared to a passive control group, produced significant improvements in sustained attention, inhibition, WM, as well as a decrease in behavioral-type ADHD symptoms after 25 training sessions, as rated by parents. These results were maintained at follow-up (six months after intervention). As this is a non-independent research, the results must be considered with caution.

#### Play attention

Steiner et al. (2011) demonstrated the effectiveness of two neuroscientific interventions for children with ADHD disorder: an NF training program (“Play attention”) and a computerized cognitive training program (“Brain Train/Captain's Log”). After an average of 23.4 sessions in their schools, parents reported an improvement in symptoms associated with this disorder which was significantly higher than that reported for the control group. In later studies, the same authors demonstrated that the effects were maintained at 6-month follow-up (Steiner et al., 2014).

#### Braingame brian

This online platform is designed to train EF and was endorsed by a randomized, double-blind, placebo-controlled trial on children with ADHD aged 8–12 years (Dovis et al., 2015). The experimental group received 25 sessions of 30–35 min each. After training, the trained group significantly improved in EF trained skills (NT). No FT on behavior or long-term effects were found.

#### ACTIVATE™

This online platform to train attention is supported by a study which tests NT (Bikic et al., 2015). In this randomized, controlled trial with children with ADHD (aged 6–13), the results showed that the trained group (40 min per day for 6 days per week over 8 weeks at home) displayed significant improvements in the primary outcome of attention. No long-term effect was confirmed.

#### SIGUEME application

This application designed for autistic children is supported by a controlled study to test its efficacy. The study conducted by Vélez-Coto et al. (2017) involved the training of children using this application for 25 sessions of 10–15 min each. Following training, the results showed that the children improved in the areas of attention, association and categorization, and interaction (NT). Nevertheless, it must be considered that the assessment was designed by researchers.

#### TALI attention training program

This program which aims to train attention is supported by a recent study on program efficacy in children with intellectual and developmental disabilities (Kirk et al., 2017). The children were randomly assigned to a training group or to a placebo control. The trained group received 25 sessions of 20 min. Although after training no significant effects were found, scores in numeracy increased at a 3-month follow-up. It must be considered that this study only assessed FT on academic achievement.

## Discussion

The present paper highlights and summarizes the current state of BT research focused on children in recent years. It also defines different commercially available BT programs for these children by type of method or research applied to test program efficacy. This summary should be particularly useful for psychologists, educators, and parents for practical purposes. A necessary consideration is that many BT programs are commercially available for children, yet the majority have not been endorsed by empirical research results. Here we attempt to provide a better understanding of which of these programs are supported by research, including their shortcomings and suggestions for future research.

BT or CT should attempt to produce some observable brain changes. As we have found, only a few BT products that are commercially available have empirical data that support evidence of neuroplasticity. Some BT programs have shown neuroplasticity using neuroimaging techniques such as FastForWord for children with dyslexia (Temple et al., 2003), Teach-The-Brain in typically-developing children (Rueda et al., 2005), Cogmed for typically-developing children (Söderqvist et al., 2012; Astle et al., 2015; Barnes et al., 2016), cancer survivors (Conklin et al., 2015), and for children with ADHD (Stevens et al., 2016), WinABC in children with dyslexia (Penolazzi et al., 2010), Luminosity in cancer survivors (Kesler et al., 2011b), and those with Turner syndrome (Kesler et al., 2011b), and Focus Pocus in children with ADHD (Johnstone et al., 2017). These suggestive neural changes are meant to reflect some improvement in cognition or behavior. Regarding FT, the results are more encouraging in the clinical population than for typically-developing children, however, due to the limitations of many of the studies, further research is required. Despite this, most BT programs claim to be based on neuroplasticity, yet, the majority are not supported by sufficient empirical research. Furthermore, confirming the existence of a relationship between neuroplasticity and transfer would provide more robust results in terms of program efficacy, because the relation between neural changes and improvements in cognition or behavior is still largely unexplored.

One of the challenges for BT is not only to produce NT (improvement in a task or skill similar to the one that was trained), but FT (improvement in an untrained task or skill which may produce some significant difference in the user's daily life). Several studies have shown transfer of different available programs and in different populations. Brain Train (Captain's Log) have shown NT in children with ADHD (La Marca and O'Connor, 2016) as well as FT for ADHD symptoms (Rabiner et al., 2010; Steiner et al., 2011), yet, no long-term effects have been found. Cogmed is supported by the largest number of research studies on children and BT. This program has been tested on typically-developing children, yet the ones showing positive NT and FT results in these populations are non-independent research: NT in Pre-schoolers (Thorell et al., 2009) FT in word-decoding (Bergman-Nutley et al., 2011) and math and reading for children aged 9–11 years with long-term effects at 2 years (Söderqvist and Nutley, 2015). Despite this, independent research has found inconclusive results in children aged 9–16 years related to WM (Gibson et al., 2012) and in 12-year-olds with no transfer effects and no long-term effects (Hitchcock and Westwell, 2017). In this case, we may ask ourselves why should this program be used with general population when there is a lack of consistent results. On the other hand, Cogmed seems to have some benefits in children with ADHD: Cogmed has shown NT in ADHD or children with attention difficulties, as well as low WM (Gibson et al., 2011; Dunning et al., 2013; Hovik et al., 2013; Chacko et al., 2014; Dongen-Boomsma et al., 2014; Ang et al., 2015; van der Donk et al., 2015; Roberts et al., 2016), FT over inhibition and reasoning through non-independent research (Klingberg et al., 2002, 2005) academic performance: math (Holmes et al., 2009; Dahlin, 2013; Holmes and Gathercole, 2014), math and reading (Egeland et al., 2013) on central EF (Holmes et al., 2010), EF(Bigorra et al., 2016), ADHD symptoms (Beck et al., 2010; Bigorra et al., 2016), and reduced off-task symptoms while performing tasks. (Green et al., 2012). Nevertheless, only a few of these studies have shown long-term effects on NT (Dunning et al., 2013; Hovik et al., 2013; van der Donk et al., 2015; Roberts et al., 2016), and on FT after 4 months (Beck et al., 2010), 6 months (Holmes et al., 2009; Bigorra et al., 2016), and 7–8 months (Dahlin, 2013; Egeland et al., 2013). It seems that the majority of studies do not demonstrate long-term effects of training. NT of Cogmed has been also shown in children with special needs (Partanen et al., 2015) with effects after 4 months (Bennett et al., 2013). Despite this, the authors of these studies did not find FT. Two studies have found FT on reading or instruction comprehension (Soderqvist et al., 2012) with long-term effects after 7 months (Dahlin, 2011). In children with language disabilities or hearing problems, there are two attempts to demonstrate the efficacy of Cogmed, however, the studies have not been properly randomized and controlled. NT has been shown to occur (Holmes et al., 2015) as well as some benefits over language skills related to WM, and was maintained at a 6-month follow-up (Kronenberger et al., 2011).

With regard to children at risk of developing learning difficulties, for children born preterm, a few studies have been conducted recently, especially on preschoolers, which showed NT and FT to some language skills related to WM (Grunewaldt et al., 2013) and FT to other domains related to WM, such as facial memory and narrative memory, which were preserved after 7 months of treatment (Grunewaldt et al., 2016). In the same population, Lee et al. (2016) only found the NT effect of Cogmed and no other effects on attention or behavior, mirroring the findings of previous authors. Finally, in adolescents, NT has been demonstrated and maintained after 7 months (Løhaugen et al., 2011), yet no FT has been provided. In children with low SES, there is evidence for NT (Mezzacappa and Buckner, 2010) as well as for FT on self-regulation and pre-literacy skills (Foy and Mann, 2014), yet no long-term effects were shown. Therefore, at this stage, the results for this at-risk group are inconclusive.

Diseases which may impact cognition have also come under the scope of WM training, such as cancer, epilepsy, and brain damage. The results for cancer patients seem to be inconclusive. Using samples within a wide age range from children to adolescents, NT was found by Conklin et al. (2015) as well as FT on processing speed and attention gains maintained at a 6-month follow-up; nevertheless, with a similar sample, Hardy et al. (2013) found NT and parental reports of fewer learning problems, but the results were not maintained at a 3-month follow-up. Furthermore, a wide age range has been studied for children with epilepsy and only NT has been found (Kerr and Blackwell, 2015) with maintenance after 3 months (Fuentes and Kerr, 2017). Finally, in terms of brain damage, only a few NT effects have been demonstrated in preteens and teens, yet, these were not maintained at 1-year post-intervention (Eve et al., 2016). Adaptive training is more effective than non-adaptive (as in previous findings). In a study by Phillips et al. (2016), adaptive training was shown to produce some benefits in reading (but not math) and was maintained after 3 months. In this last study, a passive control group should be added to better interpret results.

Finally, encouraging results have been found for children with behavioral problems, especially for teenagers and in combination with other techniques. Some results have shown NT at maintenance and at a 3-month follow-up, however gains of FT on IQ, inhibition, anxiety, attention and emotional symptoms were not maintained at follow-up (Roughan and Hadwin, 2011). Treatment combinations have yielded better results and maintenance, for instance, on preschoolers; using Cogmed in combination with other techniques (social skills, self-regulation strategies) benefits WM (NT) as well as other FT (academic achievement, emotion knowledge, emotion regulation, and executive functioning) maintained at 6 months (Graziano and Hart, 2016). In this case, as Cogmed is part of a wider treatment, we cannot directly attribute improvement in dependent variables to the program. Finally, it seems that Cogmed may be as beneficial as traditional treatment for teenagers (with a focus on anxiety reduction and self-control improvement), and demonstrated maintenance at a 4-month follow-up (Hadwin and Richards, 2016). Focus Pocus, apart from its neuroplasticity results in ADHD children (Johnstone et al., 2017), has demonstrated efficacy in FT on ADHD symptoms maintained after a 6-month intervention (Johnstone et al., 2012); nevertheless, those studies are non-independent and the results need replication in independent research. In another NF intervention, Play Attention has shown some FT on ADHD symptoms (Steiner et al., 2011) and long-term effects (6 months) on children ADHD (Steiner et al., 2014). Braingame Brian has shown NT in children with ADHD (Dovis et al., 2015), but not FT or long-term effects. As this platform is quite new, future research will be needed to clarify its benefits. The same may be said about ACTIVATE™ where NT have been also found in ADHD children, but with no other results (Bikic et al., 2015). Finally, we have included two touchscreen intervention products: SIGUEME has shown positive results regarding NT with autistic children (Vélez-Coto et al., 2017). In contrast, for the TALI attention training program, another touchscreen intervention, the research provided only non-significant improvement in children with intellectual and developmental disability (Kirk et al., 2017).

A number of other programs have been supported by empirical research presented at professional conferences, and we hope to find further research and publications on these programs in future major scientific reviews. For instance, Arrowsmith, one of the best-known computer-based interventions for children with specials needs, is supported by an intervention trial conducted with children with learning disabilities, showing NT after treatment (Fitzer et al., 2014; Kubas et al., 2014). In this case, despite the fact that it has been on the market for several years, there is little evidence on it efficacy. Uno brain is supported by empirical research, presented in conferences, on an adult population (Fernández-Sánchez et al., 2013a,b) and on children with ADHD (Fernández-Sánchez et al., 2014). The results of this study seem encouraging because they report NT and FT over ADHD symptoms. Nevertheless, other well-known platforms and computer-based interventions, such as Cognifit, Brain Master, Happy Neuron, Neuron UP, Fit Brains, Sincrolab Kids, Gomins application, Beebrite Edu, Identifor, and the Nexxo application still lack published empirical research conducted with child populations. Independent randomized controlled trials with proper follow-ups will aid us to clarify the efficacy of these emerging computer-based interventions for children.

In general, we have found some limitations of commercially available BT products: (1) lack of scientific validity of many programs designed to train specific brain skills; (2) only 10 studies (14.2%) have been found to demonstrate neuroplasticity yet the majority of BT platforms claim to be based on these concepts without providing any scientific data; (3) only 36 of a total of 70 (51.4%) studies have shown FT, and, only 11 of them (15.7%) maintained FT at follow-up, which may lead to question the efficacy of BT products in the long term, and, finally, (4) lack of accessibility such as high prices, which make these products accessible to developed countries, but not worldwide.

Considering the methodological designs in the total of 70 published articles included in this review, we found: (1) fewer of half of them (30 or 42.8%) were randomized-controlled; (2) only 13 (18.6%) included an active control group and only 2 (2.9%) included 3 groups (experimental group/active control group/passive control group); (3) more than half of them, (38 or 54.3%) included follow-ups; (4) a double-blind design was not common, present in only 9 studies (12.9%); and finally (5), a minority of studies were non-independent (11 or15.7%). Considering the research limitations discovered, we consider that further research is needed to scientifically validate the new BT programs available on the market, through double-blind randomized controlled trials, which include a passive control group and active control group, in addition to proper follow-up assessments. As we have seen, the majority of studies do not include an active control group and any follow-up beyond 6 months. Furthermore, a combination of neuroimaging techniques and psychometrical tools could be a robust method to demonstrate neuroplasticity and transfer effects to everyday life. For research designs we recommend that researches review criteria proposed by the IoM report (Mahncke and Merzenich, 2015) about how to evaluate a BT program. It is necessary to consider some study limitations such as sample sizes, lack of tasks to evaluate transfer (Cortese et al., 2015), as well as the individual differences of the participants and their motivations. Thus, some authors propose different study designs to test programs including micro-trials and single-case studies (Granic et al., 2014).

Having seen the limitations of many BT programs to produce FT and long-lasting effects, together with the methodological research limitations, a combination of treatments might potentially be more profitable; i.e., using BT as part of a wider treatment. Thus, programs which involve not only BT but also other strategies, thereby offering a treatment combination, may be more beneficial for some populations, such as children with behavioral problems, and produce more sustainable effects, as suggested by Graziano and Hart (2016), or in children with special needs, as indicated by Partanen et al. (2015). These findings support the idea that a combination of methods may be more profitable to implement and maintain cognitive and behavioral improvements over time. Future research should aim to clarify whether a combination of strategy implementation and programs would have a more significant and sustainable effect.

Despite finding the benefits of BT or a treatment combination, some authors remain unconvinced by the difficulties BT programs reported here (e.g., reaching FT and long-lasting effects), and claim that other activities that form part of children's natural environment, such as video games, music, and sports, show a more reasonable and generalized effect (Green and Bavelier, 2008). These authors emphasize that these activities are natural forms of training in which several skills are practiced in parallel. If there are common activities that foster children's skills, should BT be incorporated for typically-developing children? Is it necessary to use a BT program to improve cognitive skills in typically-developing children while there are other activities in their everyday lives that seem to benefit them as well? Why should we aim to improve children's abilities beyond usual child development?

The results obtained for child populations are controversial because there is a large proportion of non-independent research. Regarding neuroplasticity, independent research has yielded positive results (Rueda et al., 2005; Astle et al., 2015; Barnes et al., 2016), and on NT (Gibson et al., 2012; Hitchcock and Westwell, 2017) and FT (Fälth et al., 2016). Non-independent research has produced better results in these populations regarding transfer or long-term effects (Temple et al., 2003; Thorell et al., 2009; Bergman-Nutley et al., 2011; Söderqvist et al., 2012; Bergman-Nutley and Klingberg, 2014; Söderqvist and Nutley, 2015). Despite the fact that BT marketing is aimed at the general population, considering the results, we believe that BT research should contribute to validate programs as treatment tools for neurologically impaired patients, such as children with ADHD, learning disabilities, and behavioral problems. Further research is required to test the efficacy of BT and to ascertain for which populations it may be suitable, and what strategies can foster the efficacy and long-term effects of CT.

### Permission to reuse and copyright

Permission must be obtained for use of copyrighted material from other sources (including the web). Please note that it is compulsory to follow figure instructions.

### Tables

Tables should be inserted at the end of the manuscript. Tables must be provided in an editable format e.g., Word, Excel. Tables provided as jpeg/tiff files will not be accepted. Please note that very large tables (covering several pages) cannot be included in the final PDF for reasons of space. These tables will be published as Supplementary Material on the online article page at the time of acceptance. The author will be notified during the typesetting of the final article if this is the case.

## Nomenclature

### Resource identification initiative

To take part in the Resource Identification Initiative, please use the corresponding catalog number and RRID in your current manuscript. For more information about the project and for steps on how to search for an RRID, please click here.

### Life science identifiers

Life Science Identifiers (LSIDs) for ZOOBANK registered names or nomenclatural acts should be listed in the manuscript before the keywords with the following format: urn:lsid: <Authority>:< Namespace>:<ObjectID>[:<Version>]

For more information on LSIDs please see Inclusion of Zoological Nomenclature section of the guidelines.

Supplementary Material should be uploaded separately on submission, if there are Supplementary Figures, please include the caption in the same file as the figure. Supplementary Material templates can be found in the Frontiers Word Templates file.

Please see the Supplementary Material section of the Author guidelines for details on the different file types accepted.

## Author contributions

TR-P, EP-H, and JG-M: Conception and design of the review; TR-P: Searches, analysis, and data classification; EP-H and JG-M: Document review; TR-P: Writing of the paper.

### Conflict of interest statement

The authors declare that the research was conducted in the absence of any commercial or financial relationships that could be construed as a potential conflict of interest.
